# RSV Prevention Products and Severe RSV-Associated Disease Among Infants

**DOI:** 10.1001/jamanetworkopen.2026.5695

**Published:** 2026-04-08

**Authors:** Julia C. Bennett, Elyse Bevers, Sara Chronister, Ashley McHugh, Maham Choudry, Mary Huynh, Janet A. Englund, Scott Lindquist, Michelle L. Holshue

**Affiliations:** 1 Washington State Department of Health, Shoreline; 2 Epidemic Intelligence Service, Centers for Disease Control and Prevention, Atlanta, Georgia; 3 Immunization Services Division, Centers for Disease Control and Prevention, Atlanta, Georgia; 4 Seattle Children’s Research Institute, Seattle, Washington; 5 Career Epidemiology Field Officer Program, Centers for Disease Control and Prevention, Atlanta, Georgia

## Abstract

**Importance:**

A long-acting monoclonal antibody (nirsevimab) for use in infants aged 7 months or younger and an antenatal respiratory syncytial virus (RSV) vaccine (RSVpreF) became available in Washington state in 2023.

**Objective:**

To estimate the combined population-level impact of nirsevimab and antenatal RSV vaccine on the relative rate of RSV-associated hospitalizations and emergency department (ED) visits among infants during the first 2 respiratory seasons of use.

**Design, Setting, and Participants:**

This difference-in-diferrences cohort study used data from the Washington state syndromic surveillance program on RSV-associated hospitalizations and ED visits among infants from July 1, 2022, to June 30, 2025.

**Exposure:**

Introduction of nirsevimab for infants and antenatal RSVpreF.

**Main Outcomes and Measures:**

A difference-in-differences analysis was conducted to estimate mean relative changes in the rate of RSV-associated hospitalizations and ED visits between the period before and the period after introduction of RSV prevention products for children aged 7 months or younger (treatment group) beyond that for children aged 8 to 24 months (comparison group) using negative binomial models. Relative changes were estimated separately for July 1, 2023, to June 30, 2024, and July 1, 2024, to June 30, 2025, vs July 1, 2022, to June 30, 2023.

**Results:**

During July 2022 to June 2025, there were 16 775 RSV-associated hospitalizations and ED visits among children aged 24 months or younger in Washington state (median age, 10 months [IQR, 4-19 months]; 9228 male [55%]). The median age increased from 9 months (IQR, 3-19 months) during 2022-2023 to 12 months (IQR, 6-21 months) during 2024-2025. During the first year of limited use of RSV prevention products (2023-2024), estimates did not indicate a significant decrease in the rate of RSV-associated hospitalizations and ED visits among children aged 7 months or younger beyond temporal trends also observed for children aged 8 to 24 months (ratio of relative rates, 0.92 [95% CI, 0.80-1.03]). In the second year of use (2024-2025), there was an estimated 43.0% (95% CI, 32.0%-52.1%) relative decrease in the rate of RSV-associated hospitalizations and ED visits among children aged 7 months or younger beyond that for children aged 8 to 24 months. Relative changes were similar across racial groups, except for Native Hawaiian or Other Pacific Islander children. The estimated impact in 2024-2025 was 1.77 times (95% CI, 1.17-2.66 times) higher for White vs Native Hawaiian or Other Pacific Islander children.

**Conclusions and Relevance:**

In this cohort study of children aged 24 months or younger, nirsevimab and antenatal RSVpreF vaccine were associated with a reduced burden of RSV-associated hospitalization and ED visits among infants aged 7 months or younger. Results support continued use of these products in the state. Increased coverage will likely be associated with additional decreases in severe disease burden, particularly among populations at increased risk.

## Key Points

**Question:**

What is the population-level impact of infant nirsevimab and respiratory syncytial virus (RSV) antenatal vaccine on the relative rate of RSV-associated hospitalizations and emergency department (ED) visits among infants in Washington state?

**Findings:**

This cohort study including 16 775 RSV-related hospitalizations estimated a statistically significant 43.0% relative decrease in the population rate of RSV-associated hospitalization and ED visits for children aged 7 months or younger associated with RSV prevention products in the second year of use (2024-2025).

**Meaning:**

This study suggests that infant nirsevimab and antenatal RSV vaccine were associated with reduced burden of severe RSV disease among infants and supports continued use of these products in the state.

## Teaser

This cohort study estimates changes in the rate of respiratory syncytial virus (RSV)–associated hospitalizations and emergency department visits among infants during the first 2 respiratory seasons during which nirsevimab and antenatal RSV vaccine were available.

## Introduction

Respiratory syncytial virus (RSV) is an important cause of morbidity and mortality among young children.^1^ Before general population use of RSV infant monoclonal antibodies and antenatal RSV vaccine in the US, approximately 1% to 2% of infants were hospitalized with RSV in their first year of life, and almost all children were infected with RSV by 2 years of age.^2,3^

In 2023, the US Centers for Disease Control and Prevention (CDC) recommended a long-acting infant monoclonal antibody (nirsevimab) for all infants aged 7 months or younger and for a small proportion of children aged 8 to 19 months with increased risk for severe RSV disease, as well as RSV prefusion F protein–based (RSVpreF) vaccine for administration during pregnancy. These products were first used in Washington state during the autumn of 2023.^4,5^ Early postlicensure effectiveness studies have estimated nirsevimab effectiveness of 77% to 90% and RSVpreF antenatal vaccine effectiveness of 78% against RSV-associated hospitalization among US infants.^6,7,8,9^ Initial population impact data on the combined use of nirsevimab and RSVpreF vaccine in the US estimated a 28% to 43% decrease in the rate of RSV-associated hospitalization among infants aged 7 months or younger during 2024-2025 compared with the RSV seasons before the COVID-19 pandemic.^10^ This analysis seeks to estimate the combined population-level impact of infant nirsevimab and antenatal RSVpreF vaccine on the rate of RSV-associated hospitalizations and emergency department (ED) visits among infants in Washington state using syndromic surveillance data.

## Methods

### Data Source

This cohort study used data on clinically diagnosed RSV-associated hospitalizations and ED visits from the Rapid Health Information Network (RHINO), the syndromic surveillance program at the Washington State Department of Health.^11^ We included all events in the state with an RSV *International Statistical Classification of Diseases and Related Health Problems, Tenth Revision* (*ICD-10*) diagnosis code among Washington residents aged 24 months or younger during July 1, 2022, to June 30, 2025. RSV *ICD-10* diagnosis codes included the following: B97.4 (RSV as the cause of diseases classified elsewhere), J12.1 (RSV pneumonia), J20.5 (acute bronchitis due to RSV), and J21.0 (acute bronchiolitis due to RSV). Population denominators for estimating incidence rates were from the Washington State Office of Financial Management.^12^ This study was reviewed by the Washington State institutional review board and CDC and was determined to be a disease surveillance activity and therefore exempt from institutional review board review. This study was reviewed by the CDC and was conducted consistent with federal law and CDC policy (45 C.F.R. part 46, 21 C.F.R. part 56; 42 U.S.C. Sect. 241[d]; 5 U.S.C. Sect. 552a; 44 U.S.C. 319 Sect. 3501 et seq). Results reporting adhered to the Strengthening the Reporting of Observational Studies in Epidemiology (STROBE) reporting guideline.^13^

July 2022 to June 2023 was defined as a prevaccine respiratory season. July 2023 to June 2024 (availability of RSV prevention products began in October 2023) and July 2024 to June 2025 (second year of routine RSV prevention product use) were defined as postvaccine respiratory seasons. Data before 2020 were excluded from analysis because of a large increase in the availability and use of RSV testing beginning in 2021.^14^ Data during 2020 to 2021 were excluded because of a disruption in RSV trends as a result of nonpharmaceutical interventions implemented to reduce transmission of SARS-CoV-2.^15^ In addition, data during 2021 to 2022 were excluded because of historically unusual RSV trends as the virus reemerged after lifting of nonpharmaceutical interventions.^16^

### Statistical Analysis

We used a controlled quasi-experimental study design to estimate the population impact of the combined introduction of infant nirsevimab and antenatal RSVpreF vaccine with the relative rate of RSV-associated hospitalizations and ED visits among age-eligible infants (≤7 months) in Washington compared with children aged 8 to 24 months. The case age was the age at the time of illness. We conducted a difference-in-differences analysis to estimate the mean relative change in the rate of RSV-associated hospitalizations and ED visits between the period before and the period after the introduction of RSV prevention products for children aged 7 months or younger (ie, the treatment group that is age eligible for routine receipt of monoclonal antibodies) beyond the change for children aged 8 to 24 months (ie, the comparison group) using negative binomial models. Relative changes were estimated separately for July 1, 2023, to June 30, 2024, and July 1, 2024, to June 30, 2025, compared with July 1, 2022, to June 30, 2023. The models included a population denominator offset and random effects for the intercept and effect estimate by county to account for clustering and to estimate county-specific impact estimates. As there are historically disparities in pediatric RSV burden by race,^17^ as a secondary analysis, we estimated if the impact varied by race by including an interaction term for race in a state-level model. Race was as documented in electronic medical records, and categories included American Indian or Alaska Native, Asian, Black or African American, Native Hawaiian or Other Pacific Islander, White, multiracial, other, and unknown. No additional information was available for the multiracial, other, and unknown race categorizations. All analyses were conducted in R, version 4.4.1 (R Project for Statistical Computing). All *P* values were from 2-sided tests and results were deemed statistically significant at *P* < .05.

## Results

During July 2022 to June 2025, a total of 16 775 RSV-associated hospitalizations and ED visits among children aged 24 months or younger (median age, 10 months [IQR, 4-19 months]; 9228 male [55%] and 7547 female [45%]; 348 American Indian or Alaska Native [2%], 738 Asian [4%], 980 Black or African American [6%], 624 Native Hawaiian or Other Pacific Islander [4%], 7662 White [46%], 116 multiracial [1%], 4510 other race [27%], and 1797 unknown other race [11%]) were captured by syndromic surveillance in Washington state (Table 1). The median age increased from 9 months (IQR, 3-19 months) during 2022 to 2023 to 10 months (IQR, 4-18 months) during 2023 to 2024 to 12 months (IQR, 6-21 months) during 2024 to 2025. Overall, 6840 cases (41%) were among children aged 7 months or younger, and 9935 (59%) were among children aged 8 to 24 months. Most cases were among patients who were seen during ED visits only (13 589 [81%]). A total of 2589 cases (15%) were among patients admitted from the ED and 597 (4%) were among patients directly admitted to the hospital. The most common RSV *ICD-10* diagnosis code was “acute bronchiolitis due to RSV” (13 599 [81%]), followed by “RSV as the cause of diseases classified elsewhere” (2998 [18%]), “RSV pneumonia” (292 [2%]), and “acute bronchitis due to RSV” (219 [1%]).

**Table 1.** Characteristics of RSV-Associated Hospitalization and ED Visits Among Patients Aged 24 Months or Younger in Washington State by Respiratory Season

Characteristic	July 2022-June 2023 (n = 7589)	July 2023-June 2024 (n = 4806)	July 2024-June 2025 (n = 4380)	Total (N = 16 775)
Age, No. (%)				
Median (IQR), mo	9 (3-19)	10 (4-18)	12 (6-21)	10 (4-19)
≤7 mo	3416 (45)	1998 (42)	1426 (33)	6840 (41)
8-24 mo	4173 (55)	2808 (58)	2954 (67)	9935 (59)
Sex, No. (%)				
Male	4202 (55)	2669 (56)	2357 (54)	9228 (55)
Female	3387 (45)	2137 (45)	2023 (46)	7547 (45)
Race, No. (%)^a^				
American Indian or Alaska Native	164 (2)	87 (2)	97 (2)	348 (2)
Asian	366 (5)	200 (4)	172 (4)	738 (4)
Black or African American	420 (6)	281 (6)	279 (6)	980 (6)
Native Hawaiian or Other Pacific Islander	237 (3)	175 (4)	212 (5)	624 (4)
White	3572 (47)	2178 (45)	1912 (44)	7662 (46)
Multiracial	51 (1)	31 (1)	34 (1)	116 (1)
Other race	1990 (26)	1329 (28)	1191 (27)	4510 (27)
Unknown	789 (10)	525 (11)	483 (11)	1797 (11)
Ethnicity, No. (%)^a^				
Hispanic or Latino	2126 (28)	1346 (28)	1316 (30)	4788 (29)
Not Hispanic or Latino	4589 (61)	2911 (61)	2583 (59)	10 083 (60)
Unknown	874 (12)	549 (11)	481 (11)	1904 (11)
Hospital admission, No. (%)				
ED only	5999 (79)	3950 (82)	3640 (83)	13 589 (81)
Admitted from ED	1250 (17)	732 (15)	607 (14)	2589 (15)
Directly admitted	340 (5)	124 (3)	133 (3)	597 (4)
RSV *ICD-10* code, No. (%)^b^				
Acute bronchiolitis due to RSV (J21.0)	6194 (82)	3897 (81)	3508 (80)	13 599 (81)
RSV as the cause of diseases classified elsewhere (B97.4)	1303 (17)	883 (18)	812 (19)	2998 (18)
RSV pneumonia (J12.1)	152 (2)	67 (1)	73 (2)	292 (2)
Acute bronchitis due to RSV (J20.5)	117 (2)	54 (1)	48 (1)	219 (1)

Abbreviations: ED, emergency department; *ICD-10*, *International Statistical Classification of Diseases and Related Health Problems, Tenth Revision*; RSV, respiratory syncytial virus.

^a^ Race and ethnicity were as documented in electronic medical records. No additional information was available for the multiracial, other, and unknown race categorizations.

^b^ The same case might have more than 1 RSV *ICD-10* code.

**Figure 1.**
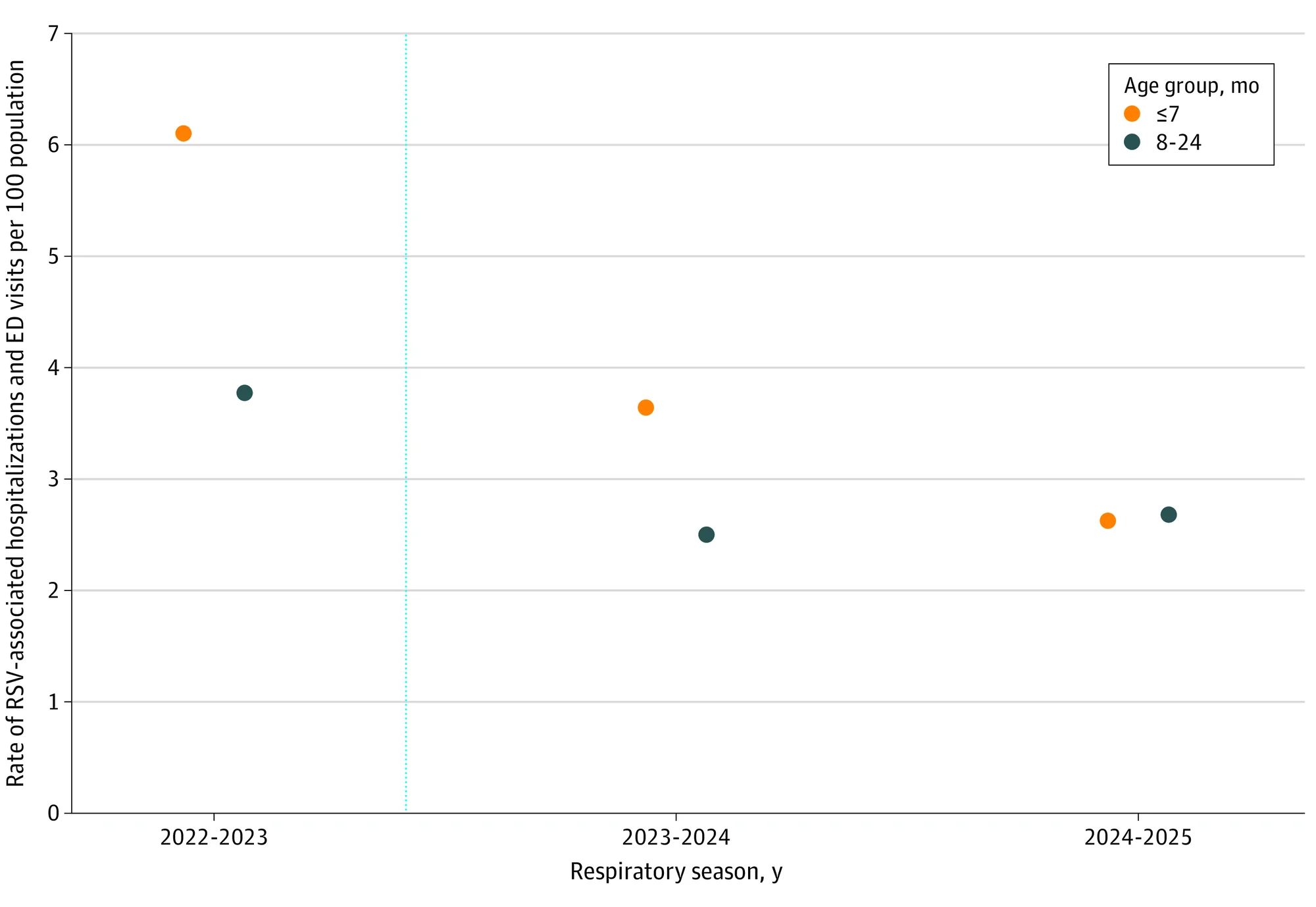
Dot Plot of Observed Annual Rate of Respiratory Syncytial Virus (RSV)–Associated Hospitalizations and Emergency Department Visits Per 100 Population Among Patients Aged 24 Months or Younger in Washington State, July 2022 to June 2025 The vertical dotted line indicates the introduction of RSV prevention products in the autumn of 2023 in Washington state: long-acting infant monoclonal antibody (nirsevimab) and prefusion F protein–based RSV vaccine for administration during pregnancy.

**Figure 2.**
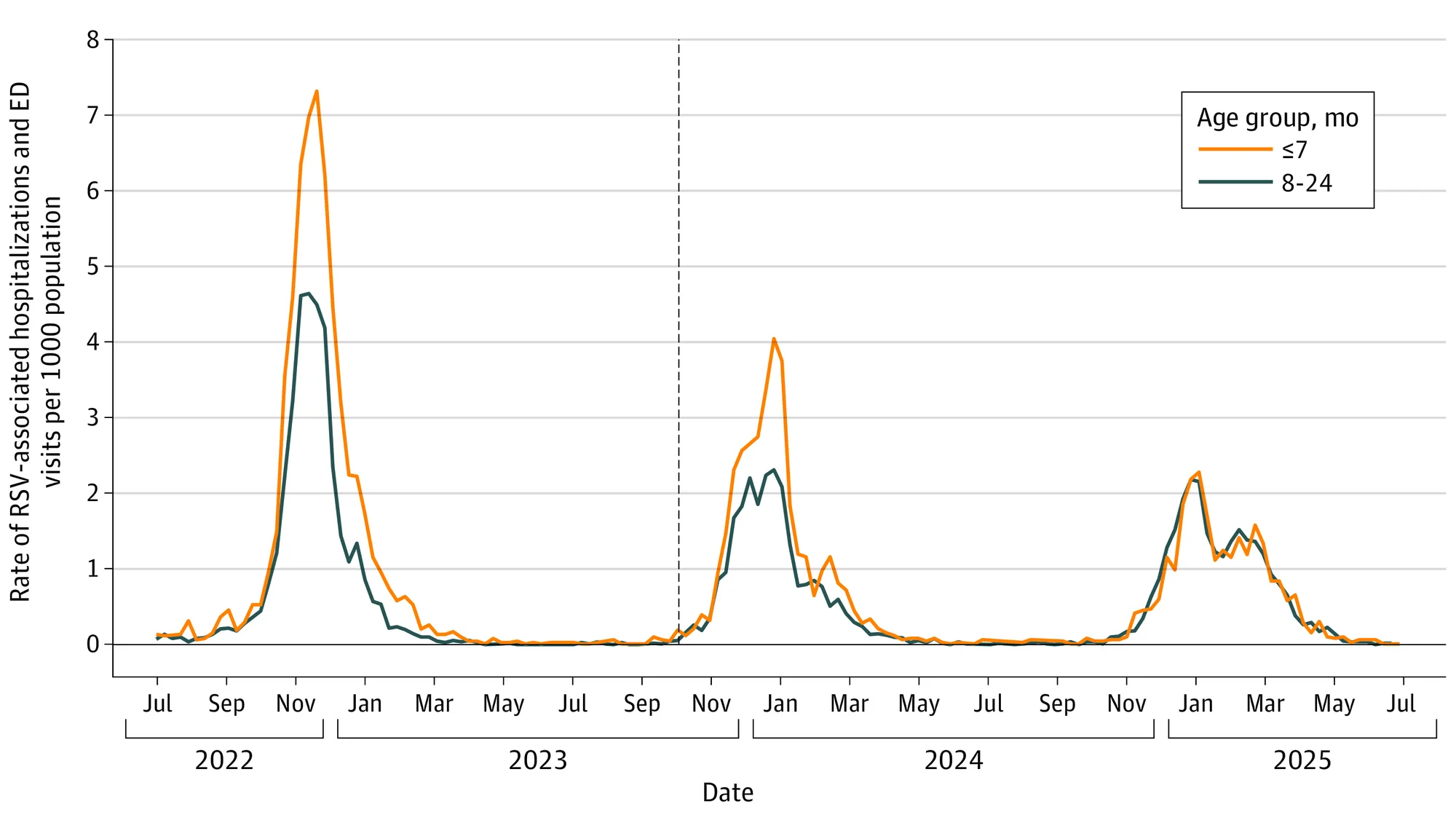
Line Graph of Observed Weekly Rate of Respiratory Syncytial Virus (RSV)–Associated Hospitalizations and Emergency Department Visits Per 1000 Population Among Patients Aged 24 Months or Younger in Washington State, July 2022 to June 2025 The vertical dashed line indicates the introduction of RSV prevention products in the autumn of 2023 in Washington state: long-acting infant monoclonal antibody (nirsevimab) and prefusion F protein–based RSV vaccine for administration during pregnancy.

**Figure 3.**
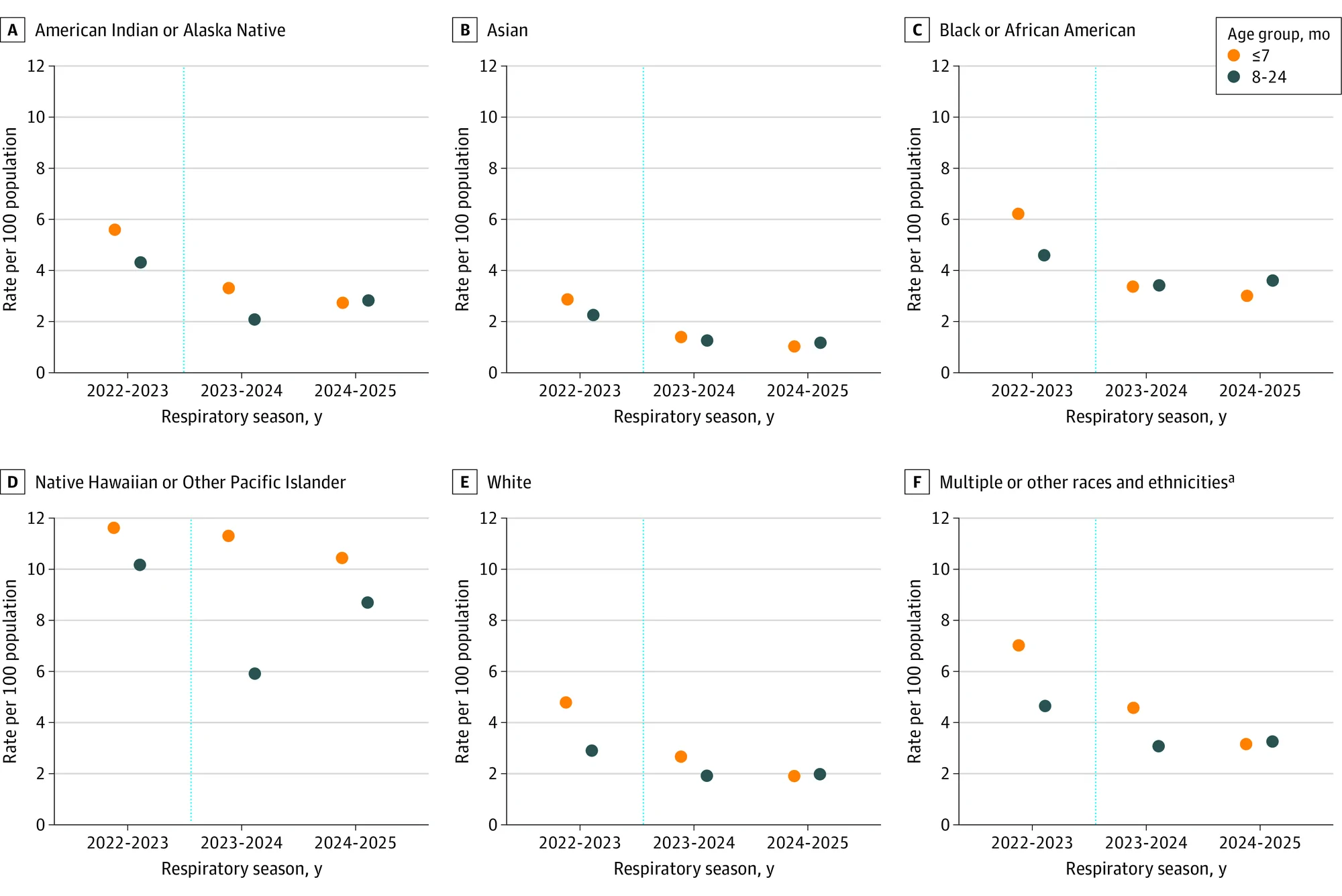
Dot Plots of Observed Annual Rate of Respiratory Syncytial Virus (RSV)–Associated Hospitalizations and Emergency Department Visits Per 100 Population for Patients Aged 24 Months or Younger in Washington State, July 2022 to June 2025 by Race and Ethnicity The vertical dotted lines indicate the introduction of RSV prevention products in the autumn of 2023 in Washington state: long-acting infant monoclonal antibody (nirsevimab) and prefusion F protein–based RSV vaccine for administration during pregnancy. ^a^Race and ethnicity were as documented in electronic medical records, and it is unknown how race and ethnicity were ascertained. The categories of multiple races and ethnicities and other races and ethnicities were combined because of this, and a lower-than-expected proportion of cases were reported as multiracial (1% vs 27% reported as other race in the medical record). Cases from persons with unknown race (1797 [11% of all cases]) are excluded. No additional information was available for the multiracial, other, and unknown race categorizations.

During 2022 to 2023, the observed annual rate of RSV-associated hospitalizations and ED visits was higher for children aged 7 months or younger (6.1/100 population) than for children aged 8 to 24 months (3.8/100 population) (Figure 1 and Figure 2). The observed annual rate decreased similarly and significantly in both age groups from the 2022 to 2023 season to the 2023 to 2024 season (3.6/100 population for children aged ≤7 months; estimated relative rate [RR], 0.61 [95% CI, 0.56-0.68]; and 2.5/100 population for children aged 8-24 months; estimated RR, 0.68 [95% CI, 0.62-0.74]) (eTable 1 in Supplement 1). During 2024 to 2025, the annual rate was similar for both age groups (2.6/100 population for children aged ≤7 months; and 2.7/100 population for children aged 8-24 months) and decreased significantly compared with 2022 to 2023 (estimated RR, 0.40 [95% CI, 0.34-0.46] for children aged ≤7 months; and 0.69 [95% CI, 0.64-0.76] for children aged 8-12 months) (Table 2). Across age groups and years, observed annual rates of RSV-associated hospitalizations and ED visits varied by race and were highest among Native Hawaiian or Other Pacific Islander children (9.2/100 person-years), followed by children with multiple races or another race (4.1/100 person-years), Black or African American children (4.0/100 person-years), American Indian or Alaska Native children (3.4/100 person-years), White children (2.6/100 person-years), and Asian children (1.6/100 person-years) (Figure 3).

**Table 2.** Estimated Relative Changes in the Rate of RSV-Associated Hospitalizations and ED Visits Among Patients Aged 24 Months or Younger in Washington State

Age	Rate of RSV-associated hospital and ED visits per 100 population^a^		Relative rate^a^
	Pre-RSV prevention products (2022-2023)	Post-RSV prevention products (2024-2025)	
≤7 mo	7.46 (6.40-8.70)	2.95 (2.47-3.52)	0.40 (0.34-0.46)
8-24 mo	4.68 (4.02-5.45)	3.24 (2.78-3.79)	0.69 (0.64-0.76)
Relative rate	1.59 (1.46-1.73)	0.91 (0.78-1.06)	0.57 (0.48-0.68)

Abbreviations: ED, emergency department; RSV, respiratory syncytial virus.

^a^ Negative binominal regression was used to estimate rates, relative rates, and 95% CIs.

During the first year of routine use of RSV prevention products (2023 to 2024) compared with 2022 to 2023, our estimate did not indicate a significant relative decrease in the rate of RSV-associated hospitalizations and ED visits among children aged 7 months or younger beyond the relative decrease that was estimated for children aged 8 to 23 months (RR, 0.92 [95% CI, 0.80-1.03]) (eTable 1 in Supplement 1). During the second year of routine use of RSV prevention products (2024 to 2025) compared with 2022 to 2023, we estimated on average, in Washington, a 43.0% (95% CI, 32.0%-52.1%; *P* < .001) relative decrease in the rate of RSV-associated hospitalizations and ED visits among children aged 7 months or younger beyond the relative decrease that was estimated for children aged 8 to 24 months (Table 2; eTable 2 in Supplement 1). That is, we estimated a 43.0% population decrease in RSV-associated hospitalizations and ED visits among children aged 7 months or younger associated with the introduction of RSV prevention products, assuming that other factors between the 2 age groups were time invariant.

The estimated population impact in 2024 to 2025 was heterogeneous across counties in Washington state and ranged from a 15.5% decrease to a 57.7% decrease in RSV-associated hospitalizations and ED visits associated with RSV prevention products (eFigure in Supplement 1). For certain smaller population counties, 95% CIs were imprecise, and the impact estimate was not statistically significant. Relative changes in RSV-associated hospitalizations and ED visits were similar across racial groups, with the exception of Native Hawaiian or Other Pacific Islander persons. The estimated population impact in 2024 to 2025 was 1.77 times (95% CI, 1.17-2.66 times; *P* = .01) greater for White children aged 7 months or younger compared with Native Hawaiian or Other Pacific Islander children of the same age.

## Discussion

During the second year of routine use of infant nirsevimab and antenatal RSVpreF vaccine, we estimated a 43% population-level decrease in the rate of RSV-associated hospitalizations and ED visits among children aged 7 months or younger in Washington state associated with RSV prevention products. No population-level impact was detected during the first year of use, when uptake of RSV prevention products was limited. This study used routinely collected syndromic surveillance data to estimate impact estimates that are generalizable for the entire state of Washington. Our population-level impact estimate is similar to that from a multistate US study (28%-43% decrease), although the study designs and methods vary.^10^ To our knowledge, our study is among the first studies in the US to estimate the population-level impact of the combined receipt of infant nirsevimab and antenatal RSVpreF vaccine. Uniquely, we used routinely collected syndromic surveillance data to evaluate the impact of these products on a reduction in the burden of severe RSV disease among infants in the US after only 2 respiratory seasons of use.

In the first year using RSV prevention products (2023 to 2024), uptake was affected by limited supply.^18^ An estimated 39% of infants born during October 2023 to March 2024 were immunized against RSV through either receipt of nirsevimab or antenatal vaccination.^19^ No coverage data are published for 2024 to 2025 yet, but preliminary unpublished data from the Washington State Immunization Information System suggest that statewide coverage of infant nirsevimab and antenatal RSVpreF vaccine approximately doubled between 2023 to 2024 and 2024 to 2025.

Our results support the use of RSV prevention products, and increased coverage of these products is expected to be associated with further decreases in infant disease. Efforts to increase coverage will be particularly impactful in geographic areas and among racial groups with increased risk of severe disease or a lower degree of impact thus far. In addition to the routine recommendation of nirsevimab for all infants younger than 8 months, the CDC also recommends nirsevimab for some children aged 8 to 19 months who are at increased risk for severe RSV disease and entering their second RSV season: certain children with chronic lung disease of prematurity, children with severe immunocompromise, certain children with cystic fibrosis, and American Indian or Alaska Native children.^5^ No such recommendation exists specifically for Native Hawaiian or Other Pacific Islander children. Our data suggest that additional work is needed to determine if a similar recommendation should be in place for Native Hawaiian or Other Pacific Islander children, for whom in Washington state we estimated lower impact of RSV prevention products and a persistently high burden of disease in the first and second years of life.

A new long-acting infant monoclonal antibody for infants younger than 8 months, clesrovimab, was approved by the US Food and Drug Administration^20^ and recommended by the CDC Advisory Committee on Immunization Practices^21^ in June 2025 and approved by the CDC director in August 2025.^22^ This newly approved monoclonal antibody will provide an additional option for infant protection against RSV and might be a simpler option given it has fixed-dose administration regardless of neonate weight and can be kept at room temperature for up to 48 hours (vs 8 hours for nirsevimab).^23,24,25^

### Limitations

This study has some limitations. Cases were defined using *ICD-10* diagnosis codes in the state syndromic surveillance system and were not laboratory confirmed. Thus, some cases might be misclassified as RSV disease. Also, estimates were imprecise for certain specific counties with limited populations that had sparse data. However, our results are representative of the entire state and might be more generalizable than studies with laboratory confirmation, which tend to be from large hospitals in urban settings.^10^ Second, race and ethnicity data were extracted from the medical record, and how these data were ascertained is unknown (eg, clinician observation, self-report, or other), and race and ethnicity data were missing for 11% of cases. Thus, an unknown amount of race and ethnicity data in this study are misclassified, which might affect our estimates by race, especially because racial and ethnic minority groups are more likely to have incomplete and inaccurate race and ethnicity data in medical records.^26^ Next, because nirsevimab is recommended for certain children aged 8 to 19 months who are at high risk for severe RSV disease, there might have been some impact of nirsevimab in our comparison group aged 8 to 24 months. In addition, it is hypothetically possible that children in our control group were protected through indirect vaccine effects or protection in the second year of life (2024 to 2025) for infants immunized in 2023 to 2024. However, we expect any impact on our results to be minimal and bias results toward the null because a limited proportion of children in this age group received nirsevimab (<1% annually in the state; Washington State Immunization Information System). Finally, this was an ecological study without individual-level data on protection from RSV prevention products and thus causality cannot be established.

## Conclusions

In this cohort study of infants aged 24 months or younger, infant nirsevimab and antenatal RSVpreF vaccine use in Washington state was associated with a reduced burden of RSV-associated hospitalizations and ED visits among infants aged 7 months or younger. Our data support continued use of long-acting infant monoclonal antibodies and antenatal vaccine in the state. Increased coverage of these products will likely be associated with additional decreases in severe disease burden for infants aged 7 months or younger, particularly in populations at increased risk.

## Notes

Supplement 1. **eTable 1.** Estimated Relative Changes in the Rate of Respiratory Syncytial Virus–Associated Hospitalizations and Emergency Department Visits Among Patients Aged ≤24 Months on Average in Washington State, July 2022–June 2023, Compared With July 2023–June 2024 **eTable 2.** Results of Difference in Difference Analysis Using Negative Binomial Regression Model to Estimate the Effect of Respiratory Syncytial Virus (RSV) Prevention Products on the Rate of RSV-Associated Hospitalizations and Emergency Department Visits Among Patients Aged ≤24 Months on Average in Washington State, July 2022–June 2023, Compared With July 2024–June 2025 **eFigure.** Estimated Relative Decline in the Rate of RSV-Associated Hospitalizations and Emergency Department Visits Among Children Aged 0–7 Months Beyond the Relative Decline That Was Estimated for Children Aged 8–24 Months by County in Washington State, July 2022–June 2023, Compared With July 2024–June 2025

Supplement 2.
Data Sharing Statement
