# Errors in Figure Keys

**DOI:** 10.1001/jamanetworkopen.2026.37937

**Published:** 2026-09-11

In the Original Investigation titled, “RSV Prevention Products and Severe RSV-Associated Disease Among Infants,”^1^ published April 8, 2026, there were errors in the figure keys of Figures 1 and 3. The labels indicating age 7 months or younger and age 8 to 24 months were transposed. This article has been corrected.^1^
