# Supplementary material for: RSV Prevention Products and Severe RSV-Associated Disease Among Infants
Source: JAMA Netw Open. 2026 Apr 8;9(4):e265695. doi: 10.1001/jamanetworkopen.2026.5695 (PMC13063090; doi:10.1001/jamanetworkopen.2026.5695)
Supplement: Supplement 1. — eTable 1. Estimated Relative Changes in the Rate of Respiratory Syncytial Virus–Associated Hospitalizations and Emergency Department Visits Among Patients Aged ≤24 Months on Average in Washington State, July 2022–June 2023, Compared With July 2023–June 2024 eTable 2. Results of Difference in Difference Analysis Using Negative Binomial Regression Model to Estimate the Effect of Respiratory Syncytial Virus (RSV) Prevention Products on the Rate of RSV-Associated Hospitalizations and Emergency Department Visits Among Patients Aged ≤24 Months on Average in Washington State, July 2022–June 2023, Compared With July 2024–June 2025 eFigure. Estimated Relative Decline in the Rate of RSV-Associated Hospitalizations and Emergency Department Visits Among Children Aged 0–7 Months Beyond the Relative Decline That Was Estimated for Children Aged 8–24 Months by County in Washington State, July 2022–June 2023, Compared With July 2024–June 2025 [file jamanetwopen-e265695-s001.pdf]

Supplementary Online Content

Bennett JC, Bevers E, Chronister S, et al. RSV prevention products and severe RSV-associated disease among infants. *JAMA Netw Open.* 2026;9(4):e265695. doi:10.1001/jamanetworkopen.2026.5695

eTable 1. Estimated Relative Changes in the Rate of Respiratory Syncytial Virus–Associated Hospitalizations and Emergency Department Visits Among Patients Aged ≤24 Months on Average in Washington State, July 2022–June 2023, Compared With July 2023–June 2024

eTable 2. Results of Difference in Difference Analysis Using Negative Binomial Regression Model to Estimate the Effect of Respiratory Syncytial Virus (RSV) Prevention Products on the Rate of RSV-Associated Hospitalizations and Emergency Department Visits Among Patients Aged ≤24 Months on Average in Washington State, July 2022–June 2023, Compared With July 2024–June 2025

eFigure. Estimated Relative Decline in the Rate of RSV-Associated Hospitalizations and Emergency Department Visits Among Children Aged 0–7 Months Beyond the Relative Decline That Was Estimated for Children Aged 8–24 Months by County in Washington State, July 2022–June 2023, Compared With July 2024–June 2025

This supplementary material has been provided by the authors to give readers additional information about their work.

© 2026 Bennett JC et al. *JAMA Network Open.*

eTable 1. Estimated Relative Changes in the Rate of Respiratory Syncytial Virus–Associated Hospitalizations and Emergency Department Visits Among Patients Aged ≤24 Months on Average in Washington State, July 2022–June 2023, Compared With July 2023–June 2024

Age	Rate of RSV-associated hospital and emergency department visits per 100 population		Relative Rate
	Pre-RSV prevention products (2022–2023)	Post-RSV prevention products (2023–2024)	
0–7 months	7.83 (6.70, 9.15)	4.81 (4.12, 5.60)	0.61 (0.56, 0.68)
8–24 months	4.89 (4.19, 5.70)	3.31 (2.83, 3.87)	0.68 (0.62, 0.74)
Relative Rate	1.60 (1.47, 1.75)	1.45 (1.05, 1.32)	0.92 (0.80, 1.03)

Abbreviation: RSV, respiratory syncytial virus.
Negative binominal regression was used to estimate rates, relative rates, and 95% CIs.

© 2026 Bennett JC et al. *JAMA Network Open.*

eTable 2. Results of Difference in Difference Analysis Using Negative Binomial Regression Model to Estimate the Effect of Respiratory Syncytial Virus (RSV) Prevention Products on the Rate of RSV-Associated Hospitalizations and Emergency Department Visits Among Patients Aged ≤24 Months on Average in Washington State, July 2022–June 2023, Compared With July 2024–June 2025

Statistical model: $y_{jt} = \beta_0 + \beta_1 post_{jt} + \beta_2 treat_{jt} + \beta_4 (post_j * treat_{jt}) + \epsilon_j$

Where,
y_{jt} = number of RSV-associated hospitalization and emergency department visits in county j at time t
$post_{jt}$ = indicator for time before introduction of RSV prevention products (0) or after introduction of RSV prevention products (1)
$treat_{jt}$ = indicator for age 0–7 months (intervention group, 1) or age 8–24 months (control group, 0)

Model Coefficient	Rate or Relative Rate (RR) (95% CI)	*P-Value*
Mean rate of RSV-associated hospitalizations and emergency department visits (per 100 population) preintroduction of RSV prevention products (2022–2023) for children aged 8–24 months on average in Washington (β_0)	4.68 (4.02, 5.45)	<0.0001
RR comparing the rate of RSV-associated hospitalizations and emergency department visits pre- (2022–2023) to postintroduction (2024–2025) of RSV prevention products for children aged 8–24 months (control group) on average in Washington (β_1)	0.69 (0.64, 0.76)	<0.0001
RR comparing the rate of RSV-associated hospitalizations and emergency department visits for children aged 0–7 months (treatment group) to children aged 8–24 months (control group) preintroduction of RSV prevention products (2022–2023) on average in Washington (β_2)	1.59 (1.46, 1.73)	<0.0001
RR comparing the RR from pre- (2022–2023) to postintroduction (2024–2025) of RSV prevention products for children aged 0–7 months (treatment group) to that for children aged 8–24 months (control group) on average in Washington (β_3)	0.57 (0.48, 0.68)	<0.0001

© 2026 Bennett JC et al. *JAMA Network Open.*

eFigure. Estimated Relative Decline in the Rate of RSV-Associated Hospitalizations and Emergency Department Visits Among Children Aged 0–7 Months Beyond the Relative Decline That Was Estimated for Children Aged 8–24 Months by County in Washington State, July 2022–June 2023, Compared With July 2024–June 2025

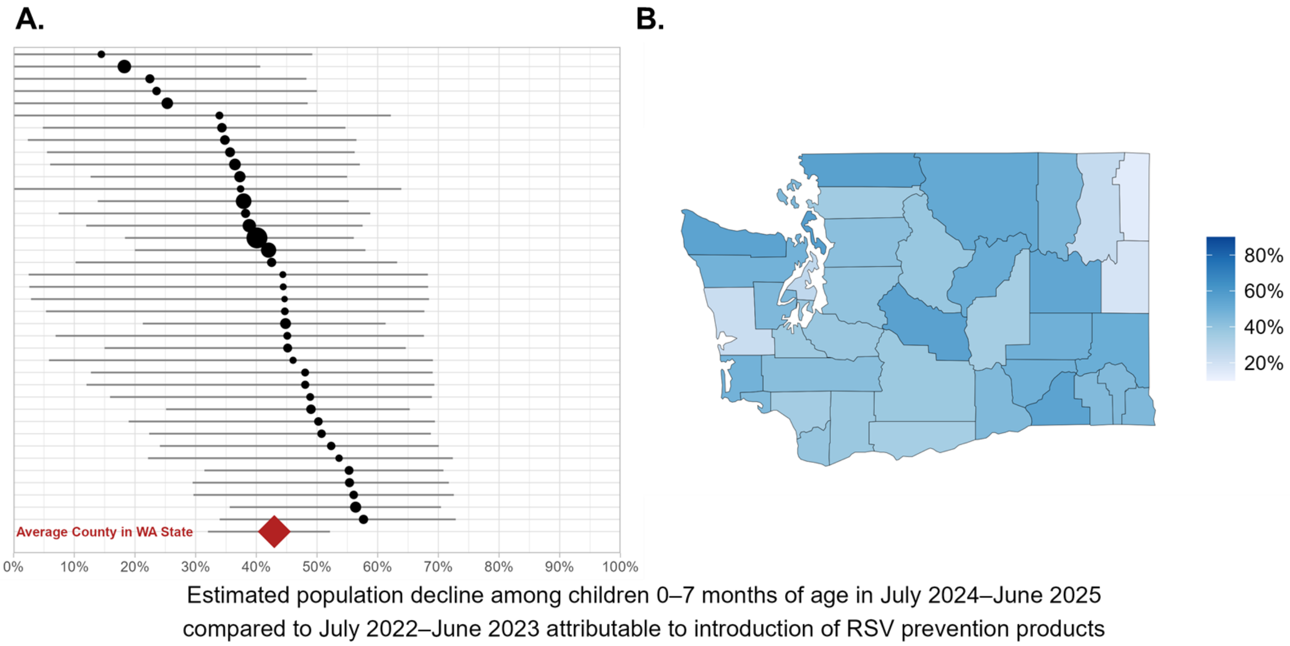

Panel A: each black point with 95% confidence bounds on the y-axis shows the effect estimate for an anonymized county in Washington State. Points are scaled by the total population of the county. Confidence bounds extending <0% not shown on the plot. The overall effect estimate on average in Washington State is shown in the red diamond at the bottom of the plot.

© 2026 Bennett JC et al. *JAMA Network Open.*
