# Supplementary material for: RSV Prevention Products and Severe RSV-Associated Disease Among Infants
Source: JAMA Netw Open. 2026 Apr 8;9(4):e265695. doi: 10.1001/jamanetworkopen.2026.5695 (PMC13063090; doi:10.1001/jamanetworkopen.2026.5695)
Supplement: Supplement 2. — Data Sharing Statement [file jamanetwopen-e265695-s002.pdf]

Data Sharing Statement

Bennett. RSV Prevention Products and Severe RSV-Associated Disease Among Infants.
JAMA Netw Open. Published April 08, 2026. doi:10.1001/jamanetworkopen.2026.5695

Data
Data available: No

Additional Information
Explanation for why data not available: Data supporting this study are owned by the Washington State Department of Health and are not available to the general public due to state laws governing the protection of confidential health information. Anonymized, aggregate data may be available upon request. To inquire about the data, please contact RHINO@doh.wa.gov.
